# Increased expression of H19/miR‐675 is associated with a low fat‐free mass index in patients with COPD

**DOI:** 10.1002/jcsm.12078

**Published:** 2016-01-05

**Authors:** Amy Lewis, Jen Y. Lee, Anna V. Donaldson, S. Amanda Natanek, Srividya Vaidyanathan, William D.‐C. Man, Nicholas S. Hopkinson, Avan A. Sayer, Harnish P. Patel, Cyrus Cooper, Holly Syddall, Michael I. Polkey, Paul R. Kemp

**Affiliations:** ^1^ Molecular Medicine SectionNational Heart and Lung Institute, Imperial CollegeSouth Kensington CampusLondonSW7 2AZUK; ^2^ National Institute for Health Research Respiratory Biomedical Research Unit at Royal Brompton and Harefield NHS Foundation Trust and Imperial CollegeLondonLondonSW3 6NPUK; ^3^ MRC Lifecourse Epidemiology UnitUniversity of Southampton, Southampton General HospitalSouthamptonSO16 6YDUK

**Keywords:** MiRNA, Cell proliferation, Regeneration, Skeletal muscle

## Abstract

**Background:**

Loss of muscle mass and strength is a significant comorbidity in patients with chronic obstructive pulmonary disease (COPD) that limits their quality of life and has prognostic implications but does not affect everyone equally. To identify mechanisms that may contribute to the susceptibility to a low muscle mass, we investigated microRNA (miRNA) expression, methylation status, and regeneration in quadriceps muscle from COPD patients and the effect of miRNAs on myoblast proliferation *in vitro*. The relationships of miRNA expression with muscle mass and strength was also determined in a group of healthy older men.

**Methods:**

We identified miRNAs associated with a low fat‐free mass (FFM) phenotype in a small group of patients with COPD using a PCR screen of 750 miRNAs. The expression of two differentially expressed miRNAs (miR‐675 and miR‐519a) was determined in an expanded group of COPD patients and their associations with FFM and strength identified. The association of these miRNAs with FFM and strength was also explored in a group of healthy community‐dwelling older men. As the expression of the miRNAs associated with FFM could be regulated by methylation, the relative methylation of the H19 ICR was determined. Furthermore, the proportion of myofibres with centralized nuclei, as a marker of muscle regeneration, in the muscle of COPD patients was identified by immunofluorescence.

**Results:**

Imprinted miRNAs (miR‐675 and from a cluster, C19MC which includes miR‐519a) were differentially expressed in the quadriceps of patients with a low fat‐free mass index (FFMI) compared to those with a normal FFMI. In larger cohorts, miR‐675 and its host gene (H19) were higher in patients with a low FFMI and strength. The association of miR‐519a expression with FFMI was present in male patients with severe COPD. Similar associations of miR expression with lean mass and strength were not observed in healthy community dwelling older men participating in the Hertfordshire Sarcopenia Study. Relative methylation of the H19 ICR was reduced in COPD patients with muscle weakness but was not associated with FFM. *In vitro*, miR‐675 inhibited myoblast proliferation and patients with a low FFMI had fewer centralized nuclei suggesting miR‐675 represses regeneration.

**Conclusions:**

The data suggest that increased expression of miR‐675/H19 and altered methylation of the H19 imprinting control region are associated with a low FFMI in patients with COPD but not in healthy community dwelling older men suggesting that epigenetic control of this loci may contribute to a susceptibility to a low FFMI.

## Introduction

Skeletal muscle is central to all aspects of physical function. Consequently the loss of muscle mass and strength that accompanies a range of chronic diseases impairs the quality of life of many individuals. Quadriceps muscle wasting^1^ and weakness^2^ are common complications of chronic obstructive pulmonary disease (COPD) and have been associated with reduced survival. Loss of muscle mass and function can occur even in mild COPD,^3^ yet some patients with severe COPD maintain muscle mass and function.^4^ In patients with severe disease, measures of muscle mass and strength are better predictors of mortality than FEV_1_.^1^ Pulmonary rehabilitation, an exercise‐based regimen that principally augments muscle mass and function, is an effective treatment in COPD^5^, ^6^ highlighting the impact of skeletal muscle on a patient's clinical condition.

In healthy adults muscle mass is maintained by balancing loss (through protein degradation, autophagy and apoptosis) with synthesis (through protein synthesis and satellite cell recruitment).^7^ In patients with chronic disease the relative rates of these processes are altered such that catabolic processes outweigh anabolic processes causing muscle loss. However, individuals are likely to have their own balance of anabolic and catabolic processes against which the insult of COPD is superimposed. If the balance of catabolism and anabolism is variable between individuals, it follows that individuals will differ in their response to an identical magnitude of atrophic insult. This suggestion is consistent with the observation that patients with the same degree of lung function impairment display a large variation in degree of muscle wasting. Susceptibility to muscle wasting may result from influences operating in early life as can be seen from the increased prevalence of sarcopenia in individuals who had a low birth weight.^8^, ^9^

MicroRNAs (miRNAs) are important regulators of cell phenotype involved in both normal development and disease.^10^ We have recently shown that there is a reduction in miR‐1 in the *quadriceps* muscle of COPD patients, associated with an increase in its target, HDAC4.^11^ Others have shown that changes in miRNA are important in atrophy associated with congenital myopathies,^12^ maintaining fibre type^13^ and muscle adaptation to exercise.^14^ MiRNAs exported into the circulation are also potential biomarkers for specific disease phenotypes and for following disease progression.^15^, ^16^ We have previously shown that levels of muscle‐specific miRNA are elevated in the plasma of COPD patients relative to controls.^17^

As many miRNAs are processed from RNA polymerase II dependent transcripts, miRNA levels will be affected by the same mechanisms that control mRNA production, including DNA methylation. Consistent with this suggestion, hypermethylation of the loci for miR‐9, ‐148, and ‐34c inactivates expression of these miRNAs in cancer cells.^18^, ^19^, ^20^ Other miRNAs are also expressed from imprinted regions of the genome so expression of one or other allele is suppressed often by methylation. Indeed two of the largest miRNA clusters in the human genome, one on chromosome 19 (C19MC) and one of chromosome 14 (C14MC), are imprinted with the C19MC expressed only from the paternal chromosome^21^ and the C14MC expressed from the maternal chromosome.^22^ Similarly, miR‐675 is processed from the maternally expressed lincRNA, H19. This gene is widely expressed during development, becomes down regulated in most adult tissues but is maintained in adult muscle.^23^ Consistent with a role in muscle development or maintenance, miR‐675 has been shown to inhibit myoblast proliferation and promote differentiation.^24^

Smoking, a primary cause of COPD, is known to modify DNA methylation through a number of mechanisms including the introduction of DNA breaks and nicotine dependent suppression of DNA methylase (DNMT1) expression (reviewed in^25^). Consequently changes in DNA methylation are likely to contribute to the changes in gene expression including that of miRNAs. The extent to which these effects occur in an individual will be dependent on factors including the relative activity of the DNA methylation cycle enzymes. Polymorphisms that lead to reduced activity of the DNA methylation cycle and hypomethylation of DNA are over‐represented in elite athletes suggestive of a role in muscle biology.^26^

The targeted studies we have performed previously have identified altered miRNA expression in the muscle and plasma of COPD patients.^11^, ^17^ This led us to hypothesize that the miRNA pattern would differ between patients with a low fat‐free mass index (FFMI) and those with a normal FFMI and that these differences would provide mechanistic insight into the susceptibility of some individuals with COPD to lose significant muscle mass. We compared miRNA expression profiles in samples of quadriceps muscle from COPD patients with a low FFMI and those with a normal FFMI. This analysis identified miRNAs from two imprinted regions of the genome, miR‐675 (from the maternally imprinted lincRNA H19) and C19MC miRNAs (from a paternally expressed cluster) as the most differentially expressed miRNAs. We validated the findings by analyzing the expression of miR‐675 and miR‐519a and the host gene for miR‐675 (H19) in a larger group of COPD patients and determined their association with FFMI and strength. As these miRNAs were derived from imprinted loci we next analysed relative DNA methylation on the H19 imprinting control region (ICR) in DNA isolated from the quadriceps of COPD patients. If the differences identified were just a measure of size rather than susceptibility to a loss of muscle mass we would have expected the same associations to exist in a normal population. We therefore determined the expression of these miRNAs in the quadriceps of healthy community dwelling older men participating in the Hertfordshire Sarcopenia Study. Finally to understand the contribution of regeneration to the difference between low FFMI and normal FFMI we determined the proportion of fibres with centralized nuclei in samples from COPD patients with a low or normal FFMI.

## Materials and methods

### Subjects

###### COPD cohort

Patients with COPD according to the Global Initiative in Obstructive Lung Disease (GOLD) guidelines 2004^27^ were enrolled from clinics at the Royal Brompton Hospital. Patients with a diagnosis of heart, renal, or liver failure, a systemic inflammatory or metabolic disorder or a moderate/severe exacerbation (i.e. requiring antibiotics, oral steroids, or hospitalization) in the preceding 4 weeks were excluded. Healthy age‐matched controls were recruited by advertisement. All subjects gave written informed consent and the protocol was approved by the Royal Brompton & Harefield NHS Trust Research Ethics Committee (Studies 06/Q0404/35 and 06/Q0410/54). Sixteen control subjects were recruited by local advertisement. A low FFMI was defined as below 16.0 kg/m^2^. 28

###### Physiological measurements

COPD subjects in this study form part of a larger well‐phenotyped cohort described by Natanek *et al.* 4 Measurements of lung volume, using plethysmography, carbon monoxide transfer factor, using the single breath technique (CompactLab, Jaeger, Germany) and post‐bronchodilator spirometry were performed according to ATS/ERS guidelines.^29^ Blood gas tensions were measured in arterialized capillary earlobe blood. Fat‐free mass index was calculated using bioelectrical impedance (Bodystat 1500, Bodystat, UK) measured in patients after resting supine for 10 min as described previously.^30^ Because of the availability of samples, it was not possible to work on a single set of samples. The physiological characteristics of the groups analysed are given in *Table* 1 and Supplementary Tables 1, 2, 4, and 5.

**Table 1:** Patient characteristics of the validation cohort

	Control (*n* = 16)	Normal FFMI (*n* = 24)	Low FFMI (*n* = 24)
Sex (M, F)	7, 9	15, 9	12, 12
Age (years)	66 ± 8	67 ± 7	64 ± 9
Smoking history^a^ (pack‐year)	0 (0, 10)	47 (35, 66)***	39 (24, 48)***
Weight^a^ (kg)	67.1 (61, 74.1)	75.1 (65.3, 83.0)	61.4 (53.5, 65.8)*, †††
BMI^a^ (kg/m^2^)	24.8 (23.5, 26.2)	26.7 (24.0, 29.7)	21.8 (19.8, 22.9)**, †††
FFMI^a^ (kg/m^2^)	16.1 (15.3, 17.2)	17.1(16.2, 17.4)	14.5 (13.4, 14.6)***, †††
FEV_1_ a (% pred)	107.6 (100.6, 112)	42.8 (25.7, 49.6)***	36.6 (27.6, 45.2)***
RVTLC	35 ± 6	58 ± 8***	60 ± 8***
TLCO^a^ (% pred)	92.3 (83.0, 98.1)	45.3 (34.8, 54.4)***	40.6 (26.7, 50.6)***
6 min walk (m)	621 ± 84	368 ± 119***	400 ± 130***
6 min walk % pred	126 ± 12	77 ± 24***	77 ± 24***
pVO_2_ a (% pred)	99 (88, 111)	51 (42, 65)***	45 (36, 51)***
SGRQ^a^	2 (0, 8)	53 (43, 60)***	56 (51, 63)***
Quadriceps MVC (kg)	34.7 ± 10.6	27.7 ± 9.8	25.7 ± 7.2**
Quadriceps MVC (% pred)	78 ± 19	66 ± 17*	61 ± 11**
Locomotion time^a^ (min/12 h)	96 (84, 127)	37 (23, 52)***	44 (26, 64)***
Movement time (as % of 12 h)	23 ± 6	12 ± 6***	13 ± 4***
Type I fibre %	54.4 ± 18.1	28.7 ± 12.1***	27.4 ± 13.2 ***
Type IIA fibre %	41.0 ± 14.0	58.5 ± 10.6***	63.6 ± 14.0***
Type IIX fibre %	2.5 ± 3.5	6.7 ± 8.2	4.8 ± 4.5

Definitions of abbreviations:

a = Not normally distributed, BMI = body mass index, FFMI = fat‐free mass index, FEV_1_ = forced expiratory volume in 1 s, RV = residual volume, TLC = total lung capacity, TL_CO_ = transfer coefficient of the lung for CO, Pa_02_ = arterial oxygen partial pressure, Pa_C02_ = arterial carbon dioxide partial pressure, pred = predicted, MVC = maximal voluntary contraction, SGRQ, St George's respiratory questionnaire. Values are means ± SD for normally distributed variables and as median (interquartile range) for variables that were not normally distributed. Significance was calculated by *t*‐test for normally distributed variables and by Mann–Whitney U‐test for variables that were not normally distributed.

* (*P* < 0.05).

** (*P ≤* 0.01).

*** (*P ≤* 0.001) low FFMI or normal FFMI vs. control.

†† (*P ≤* 0.01).

††† (*P ≤* 0.001) low FFMI vs. normal FFMI.

Quadriceps strength was determined by measuring supine isometric maximal voluntary contraction (MVC) and unpotentiated Twitch force (TwQ) as described previously^30^ and exercise capacity measured as 6 min walk distance 5 min after bronchodilator treatment (ATS 2002 guidelines^31^) as described previously. Muscle biopsy was performed by percutaneous needle biopsy of the *vastus lateralis* in the mid‐thigh of the leg that strength was tested was performed under local anaesthesia using the Bergstrom technique.^32^

###### Hertfordshire Sarcopenia study cohort

Samples from the Herts Sarcopenia Study study^33^ were used in this analysis as an additional control set to determine whether similar associations existed in a normal healthy population of older people as those identified in the COPD cohort and whether there were any associations with birth weight. Recruitment of this cohort has been described previously.^33^ The protocol was approved by the Hertfordshire Research Ethics Committee (study 07/Q0204/68), and all participants gave written informed consent. The skeletal muscle characterization carried out in this study included: body composition and lean mass by dual energy X ray absorptiometry scanning, hand‐grip strength, determined using a Jamar hydraulic dynamometer, and physical capability determined as 3m gait speed and ‘6m time to get up and go’ test. These have been previously described,^33^ and the data for the cohort used in this study are shown in *Table* S3. Muscle biopsy was performed by percutaneous needle biopsy of the *vastus lateralis* under local anaesthesia as described previously.^34^

###### TaqMan® array microRNA cards

The amplified cDNAs were quantified using Human TaqMan® Array MicroRNA Cards (Cards A and B, V.3.0 Applied Biosystems) according to the manufacturer's instructions on an ABI 7900HT thermocycler. Array cards were run under the 384 well TaqMan® Low Density Array default thermal‐cycling conditions defined by SDS 2.4 software. Once complete all cards were calibrated to a single control sample and one detection threshold set across all samples and assays using RQ manager software. The resultant Ct values were then exported and normalized to the geometric mean of U6, RNU44, and RNU48, using the ΔΔ Ct method. As variance tended to increase with miRNA intensity the data were standardized by a log transformation.

###### Assessment of mRNA and miRNA levels

Messenger RNA was extracted and quantified by quantitative real time PCR (qRTPCR) as described previously^35^ or using the allele specific primer kit for RS2075245 (Life Technologies). PCR values for H19 (obtained from 11 controls, 34 normal FFMI patients, and 22 low FFMI patients) were normalized to the geomean of RPLPO and HPRT. Primers were H19: For‐TGCTGCACTTTACAACCACTG, Rev‐TGGTGTCTTTGATGTTGGGC RPLPO: For‐TCTACAACCCTGAAGTGCTTGATATC, Rev‐GCAGACAGACACTGGCAACATT and HPRT: For‐GCTATAAATTCTTTGCTGACCTGCTG ,Rev‐AATTACTTTTATGTCCCCTGTTGACTGG. MicroRNA expression was analysed in trizol extracted RNA. RNA isolated muscle was reverse transcribed using MultiScribe™ Reverse Transcriptase with, Megaplex™ RT Primers (human pools A and B, Version 3.0, Applied Biosystems) according to the manufacturer's instructions. The reactions were terminated by heat deactivation at 85°C for 5 min and the cDNA stored at −80°C. The cDNAs were pre‐amplified using Megaplex™ PreAmp Primers (Applied Biosystems) for 12 cycles of 95°C for 15 s and 60°C for 4 min. The reaction was terminated by heating to 99.9°C for 10 min and pre‐amplified cDNA diluted by addition of 75 μL of 0.1× TE buffer pH 8.0 (Qiagen) and stored at −80°C.

###### Quantification of single microRNAs using TaqMan® probes

For the quantification of individual microRNAs, custom designed primers and probes were purchased for each test gene from Applied Biosystems, and amplification was carried out on cDNA preamplified as described above according to the manufacturer's instructions. Each reaction was performed in duplicate, and the average Ct value normalized to the corresponding geometric mean of U6, and RNU44 using the ΔΔ Ct method. RNA isolated from cells was analysed using single RT reactions. MiRNAs were quantified in 16 controls, 24 low FFMI patients, and 24 normal FFMI patients the COPD cohort and from 67 individuals in the Herts Sarcopenia Study cohort.

### Methylation analysis

DNA from muscle biopsies (for 10 controls, 14 normal FFMI patients, and 15 low FFMI patients) was extracted using the QIAamp DNA Mini kit (Life Technologies) according to manufacturer's instructions. The two resulting elutions were then pooled and precipitated. The resuspended DNA was diluted to 12.5 ng/μL in AFA fiber pre‐slit Snap‐Cap microtubes (Covaris, Life Tech) and sheared using a Covaris S220 Focused‐ultrasonicator to sonicate the DNA into 200 bp fragments using a pre‐set protocol in the SONOLAB 7.2 software (Covaris, Life Tech). DNA (1 µg) was applied to the MethylMiner Methylated DNA Enrichment kit (Life Tech) according to the manufacturer's instructions. The resulting methylation enrichment DNA was eluted in 2000 mM and 450 mM NaCl, which were then concentrated and pooled. The DNA was then quantified using the Quant‐iT PicoGreen dsDNA (Life Tech) according to manufacturer's instructions. The methylation enriched DNA was diluted to 0.1 ng/μL whilst the original (non‐enriched) DNA was diluted to 1 ng/μL; 1.5 µl of DNA was amplified in 20 µl SYBR reactions as described for cDNA using primers UBE2B: For‐CTCAGGGGTGGATTGTTGAC, Rev‐TGTGGATTCAAAGACCACGA, H19 ICR: For‐TTGGTGGAACACACTGTGATCA, Rev‐GAGCCGCACCAGATCTTCAG.

###### Immunofluorescence

Frozen 10‐µm‐thick slices from quadriceps muscle biopsies of both low FFMI (*n* = 5) and normal FFMI (*n* = 10) patients (Supporting Information, *Table* S5) were fixed in 4% PFA for 10 min at room temperature. Following fixation sections were washed in 1× PBS‐T (3× for 3 min), prior to incubation in 5% BSA‐PBS‐T solution for 30 min at room temperature. The sections were then incubated with anti‐laminin 2α antibody (1:300, AbCam) overnight at 4°C in a humidified chamber. The primary antibody solution was removed, and the sections were washed before incubation with anti‐rabbit IgG conjugated to Alexafluor‐658 (1:500, Invitrogen). The sections were incubated with the secondary antibody for 1 h at room temperature, then washed and before incubating with a DAPI solution (1:10 000) for 15 min.

###### Statistical analysis

All gene expression data were log transformed to stabilize variance and produce a normal distribution. To identify groupings within the data set principal components analysis (PCA) and hierarchical cluster analysis (HCA) were performed in Aabel (Gigawiz). The variables for HCA were scaled to unit variance, distances were calculated based on correlation coefficients, and groups were defined based on centroid linkage. Correlation analysis was performed using Pearson correlations assuming that correlations would be linear (Aabel). Differences between groups were calculated by Student's *t*‐test for normally distributed data (Excel) and by Mann–Whitney U‐test for non‐parametric data (Aabel, Gigawiz).

## Results

### Patient characteristics

miRNA profiles were determined in RNA isolated from the quadriceps of, seven patients with a low FFMI, seven patients with a normal FFMI, and seven age‐matched controls. All samples in this analysis were from males, and their physiological characteristics are shown in *Table* S1. Consistent with a diagnosis of COPD, both the low FFMI and normal FFMI patient groups had reduced FEV_1_ and TL_CO_% of predicted. *Quadriceps* strength, physical activity levels, and exercise performance were all reduced compared to controls. Both the low FFMI and normal FFMI COPD groups had a lower mean FFMI than the healthy controls. The low FFMI and normal FFMI groups did not differ in any characteristic measured including daily physical activity except, by design, FFMI, and consequently weight and BMI (Supporting Information, *Table* S1). Furthermore, there was no difference in fibre type proportion between the patient groups although both the FFMI groups had a lower proportion of type I fibres than the controls (Supporting Information, *Table* S1).

### Low FFMI patients are a distinct sub‐group of COPD patients

**Figure 1:**
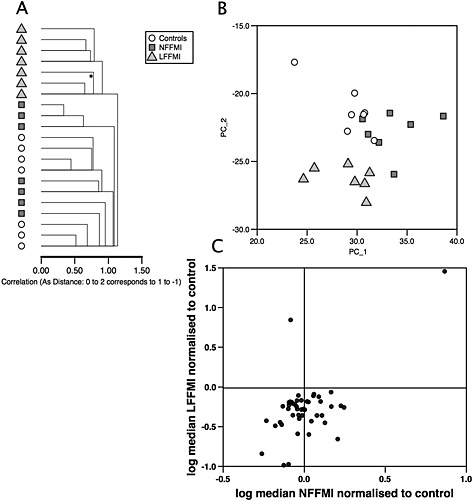
MicroRNA expression in patients with chronic obstructive pulmonary disease. Hierarchical cluster analysis (A) and principal component analysis (B) of microRNA expression in the quadriceps of low fat‐free mass index, normal fat‐free mass index patients, and controls show that the low fat‐free mass index group have a distinct pattern of microRNAs compared to normal fat‐free mass index patients and controls. Separation between the normal fat‐free mass index and control groups was weaker and had some degree of overlap. (C) Comparison of the median expression of microRNAs between low fat‐free mass index patients and controls and between normal fat‐free mass index patients and controls to identify ‘low fat‐free mass index‐associated microRNAs’. Median expression of microRNAs in the bottom right hand quadrant is lower in low fat‐free mass index patients than controls (statistically significant) but higher in the normal fat‐free mass index patients than controls (not statistically significant) whereas median expression of the microRNA in the top left hand quarter is higher in low fat‐free mass index patients than controls (statistically significant) but lower in normal fat‐free mass index patients than controls (not statistically significant).

HCA and PCA, showed that the low FFMI patients formed a distinct group based on their miRNA profile. PCA but not HCA was also able to partially segregate normal FFMI patients and controls; however, there was overlap in these two groups (*Figure* 1A and B). This result was confirmed by linear discriminate analysis, which allowed us to correctly assign the entire low FFMI group, all controls and five of the seven normal FFMI patients from their PCA scores.

### Identification of FFMI associated miRNAs in muscle

To identify miRNAs associated with a low FFMI in COPD, the expression of individual miRNAs was compared by *t*‐test between the groups in a pair wise manner. To increase stringency miRNAs associated with a low FFMI in COPD were defined as those that were statistically significantly different between low FFMI patients and controls/normal FFMI patients but not statistically different between controls and normal FFMI patients.

Forty‐seven low FFMI‐associated miRNAs were identified (Supporting Information, *Table* S6). Of these only two miRNAs were increased in low FFMI patients relative to normal FFMI patients of which miR‐675 showed the greatest fold change of any miRNA (8.5‐fold low FFMI vs. normal FFMI *P* = 0.008). The remaining 45 miRNAs were expressed at lower levels in low FFMI compared to normal FFMI patients. Median expression of all but 2 miRNAs was lower in low FFMI patients compared to controls. Comparison of miRNA expression in low FFMI and normal FFMI patients relative to controls showed that the median expression of 17 miRNAs changed in opposing directions in low FFMI vs. controls (statistically significant) compared to normal FFMI vs. controls (not statistically significant) (*Figure* 1C and *Table* S6) including miR‐675, miR‐1 (consistent with our previous study^11^), and nine miRNAs from a cluster located on chromosome 19q13.2 (C19MC).

### Muscle array validation

**Figure 2:**
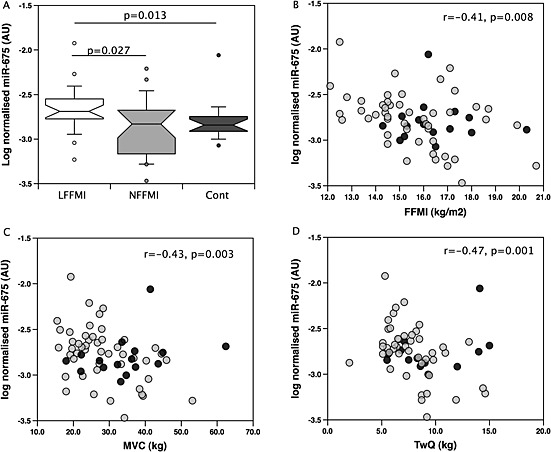
MiR‐675 is increased in low fat‐free mass index chronic obstructive pulmonary disease patients compared to normal fat‐free mass index chronic obstructive pulmonary disease patients and is associated with muscle mass and strength. The expression of miR‐675 was determined by quantitative PCR in samples of quadriceps from the validation cohort of chronic obstructive pulmonary disease patients and controls as described in Methods. MiR‐675 was elevated in patients with a low fat‐free mass index compared to controls (*P* = 0.013, A). In both patients alone and the whole cohort, miR‐675 was correlated with fat‐free mass index (*r* = −0.41, *P* = 0.008 patients alone *r* = −0.35, *P* = 0.008 patients and controls, B). In the patients alone miR‐675 was correlated with strength both voluntary (MVC, *r* = −0.43, *P* = 0.003, C) and involuntary (TwQ = −0.47, *P* = 0.001, D) measurements of strength. Patients are shown as grey circles controls as black circles.

To validate the screen, levels of miR‐675 and miR‐519a (derived from the C19MC) were quantified in an expanded cohort of muscle samples from patients including both males and females of all GOLD stages and healthy controls (controls *n* = 16, normal FFMI *n* = 24 and low FFMI *n* = 24, freshly prepared cDNA samples from five normal FFMI, one low FFMI COPD patients, and four controls from the original screen were used in this expanded study). The physiological characteristics of these groups are given in *Table* 1 and showed similar differences to those of the screen cohort. There was no difference in the expression of miR‐675 between COPD patients (as a whole group) with controls. However, median miR‐675 expression was higher in the low FFMI patients than either of the other two groups but was significantly different only compared to the controls (low FFMI vs. cont; *P* = 0.013, low FFMI vs. normal FFMI; *P* = 0.027, Bonferonni corrected *P* value for significance = 0.016). There was no difference in the expression of miR‐675 between normal FFMI patients and controls (*Figure* 2A). In the patients, miR‐675 was inversely correlated with FFMI (*r* = −0.41, *P* = 0.004, *Figure* 2B). Furthermore, miR‐675 was inversely correlated with quadriceps strength (vs. MVC, *r* = −0.43, *P* = 0.003, vs. TwQ, *r* = −0.47, *P* = 0.001, *Figure* 2C and D). MiR‐675 was not associated with lung function judged by either FEV_1_ or TL_CO_% predicted. In the controls miR‐675 was not associated with FFMI or quadriceps strength.

**Figure 3:**
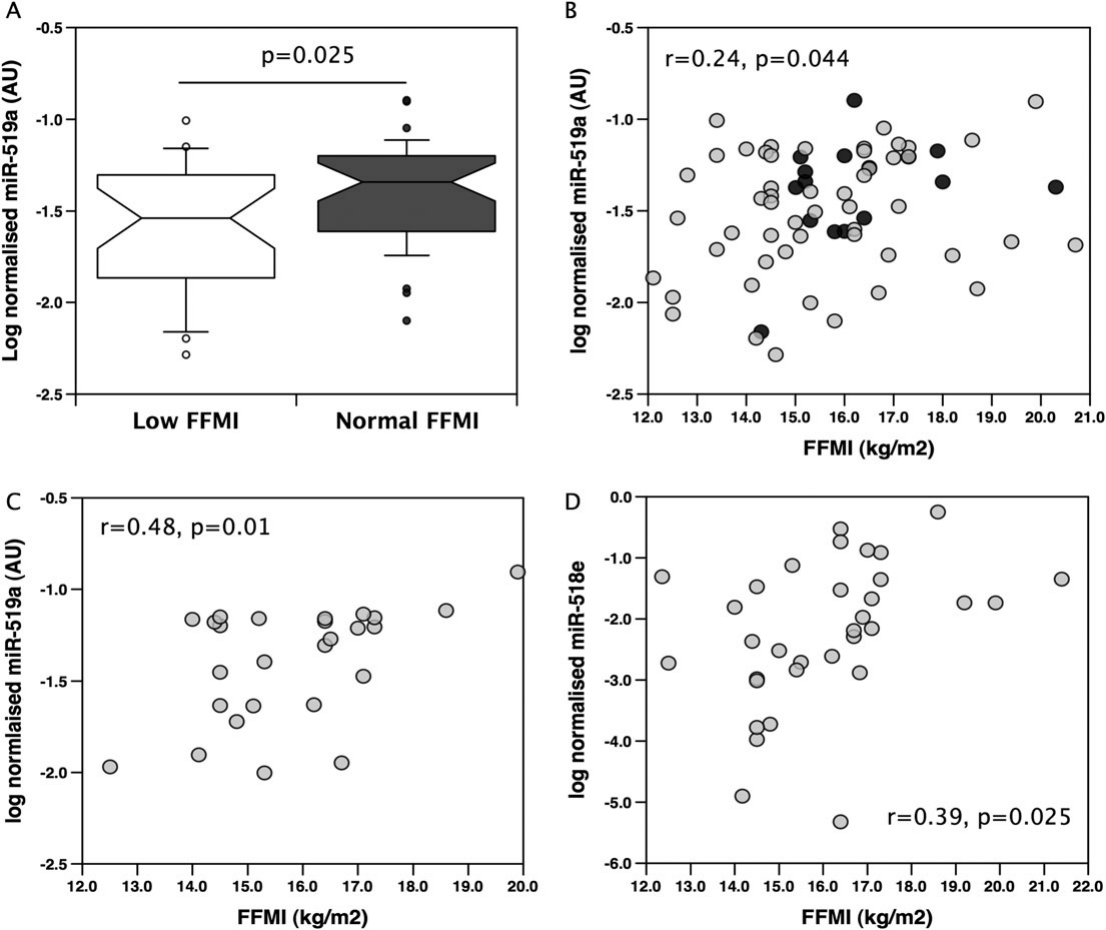
MiR‐519a is suppressed in low fat‐free mass index individuals compared to normal fat‐free mass index individuals. The microRNA expression was determined by quantitative PCR in samples of quadriceps from the validation cohort of chronic obstructive pulmonary disease patients and controls as described in Methods. In the whole cohort, miR‐519a levels were reduced in individuals with a low fat‐free mass index compared to individuals with a normal fat‐free mass index (A) and weakly correlated with fat‐free mass index (*r* = 0.29 *P* = 0.044, B). In GOLD3/4 male patients quadriceps expression of miR‐519a (C) and miR‐518e (D) were correlated with fat‐free mass index (*r* = 048, *P* = 0.01 and *r* = 0.39, *P* = 0.025 respectively). Patients are shown as grey circles controls as black circles.

There was no difference in the expression of miR‐519a between COPD patients as a whole group with controls. In the whole cohort (patients and controls together), consistent with the array, miR‐519a was lower in individuals with a low FFMI than in those with a normal FFMI (*P* = 0.025, *Figure* 3A) and weakly correlated with FFMI (*r* = 0.24, *P* = 0.044, *Figure* 3B). However, miR‐519a was not different between patients with a low FFMI compared to patients with a normal FFMI. Restricting the analysis to samples with the original criteria used for patients in the screen (male GOLD III–IV, *n* = 27) increased the association between miR‐519a and FFMI (*r* = 0.48, *P* = 0.017, *Figure* 3C). No association between miR‐519a and FFMI was found in female patients with similar disease severity (*n* = 13) raising the possibility of a sex‐specific effect. Analysis of a second miRNA from the same locus (miR‐518e) in GOLD III–IV males also showed a positive association of C19MC miRs with FFMI in these patients (*Figure* 3D).

#### H19 linc RNA expression associates with muscle mass and strength in COPD

**Figure 4:**
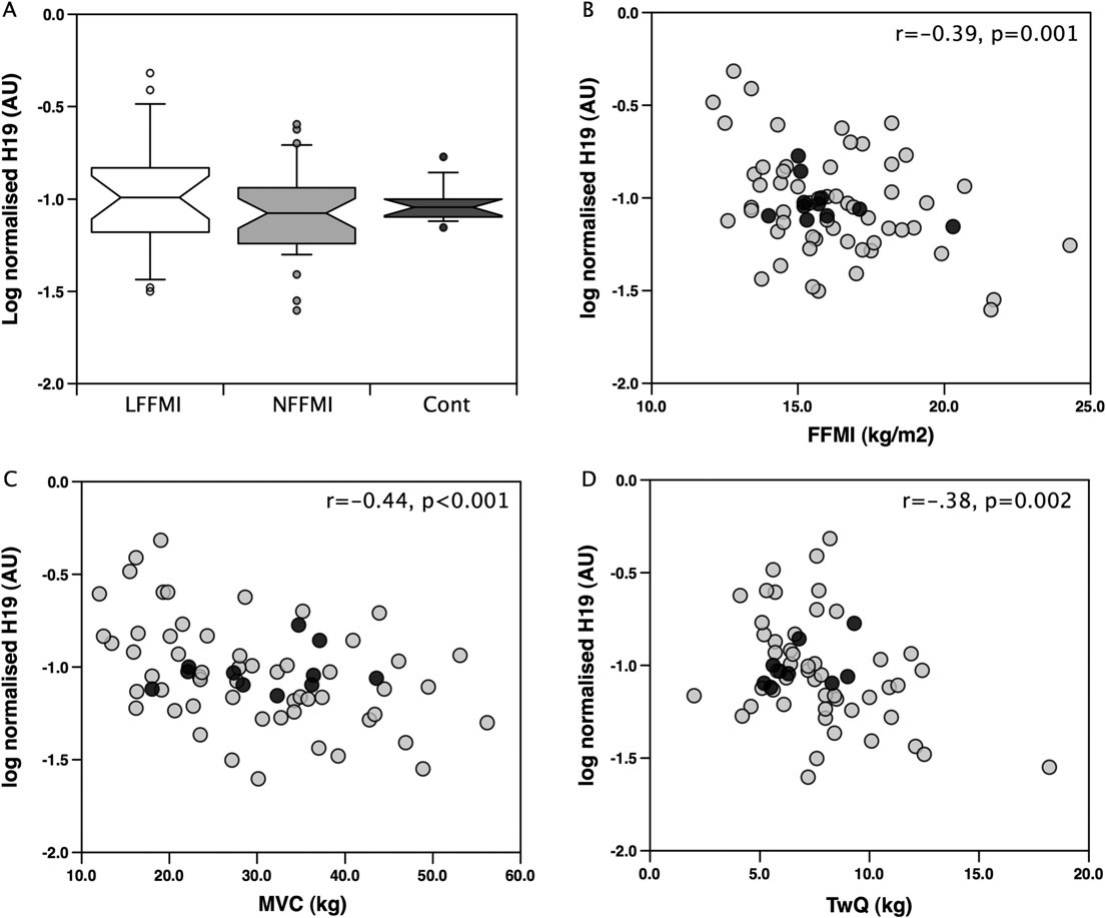
H19 is increased in low fat‐free mass index chronic obstructive pulmonary disease patients compared to normal fat‐free mass index chronic obstructive pulmonary disease patients and is associated with muscle mass and strength. The expression of H19 was determined by quantitative PCR in samples of quadriceps from the validation cohort of chronic obstructive pulmonary disease patients and controls as described in Methods. H19 not different between patient groups or controls (A). In patients H19 was correlated with fat‐free mass index (*r* = −0.385, *P* = 0.001, B) and with was correlated with quadriceps strength both voluntary (MVC, *r* = −0.44, *P* < 0.001, C) and involuntary (TwQ = −0.38 *P* = 0.002, D) measurements of strength. Patients are shown as grey circles controls as black circles.

As miR‐675 is processed from the H19 lincRNA, we determined the expression of H19. As the cDNAs for the miRNAs were generated separately from cDNAs for mRNA analysis and our samples were limited, it was only possible to obtain overlapping but distinct data sets for miR‐675 and H19. This analysis used samples from 11 controls, 34 NFFMI patients, and 22 low FFMI patients; 31 samples were included in both miRNA and mRNA analyses (see Supporting Information, *Table* S2 for the physiological characteristics). There was no difference in H19 expression between the patients and controls or between the low FFMI and normal FFMI patients (*Figure* 4). However, H19 expression was inversely associated with FFMI in the patients (*r* = −0.39, *P* = 0.001) and with quadriceps strength (vs. MVC *r* = −0.44, *P* < 0.001 and vs. TwQ *r* = −0.38, *P* = 0.007). H19 expression was also higher in those patients defined as weak based on an MVC less than 120% of their BMI (*P* = 0.01). In those patients in which we analysed both miRNA and mRNA, expression of miR‐675 and H19 showed a positive association (*r* = 0.49, *P* = 0.018, *n* = 22 patients only and *r* = 0.47, *P* = 0.007 *n* = 31 patients and controls, Supplementary Figure 1).

#### Methylation of the H19 locus is associated with muscle H19 expression and strength in COPD

To determine whether H19 was expressed from one or both alleles in adult muscle we used allele‐specific PCR for the polymorphism RS2075245, which is in exon 1 of H19, close to miR‐675. In this experiment homozygosity or mono‐allelic expression was assumed when there was difference in Cycle threshold for each of the alleles of four cycles (i.e. when one allele contributed >94% of the detectable transcript). In this analysis, expression of a single SNP variant was detected in 60% of the samples whereas expression of both variants was detected in the remaining 40%. Within the European Caucasian population the A:T allele frequency is 48:52 so that the approximately 50% of individuals are heterozygous. Therefore, in the majority of individuals both alleles were active in the muscle. However in the subjects in whom both A and T alleles were detected, one allele contributed more than 75% of the transcript in all but three cases indicating that one allele was suppressed, probably by methylation. It was not possible to determine whether the same was true from homozygous individuals. As these primers are designed for genotyping and not mRNA quantification, to verify that the method quantified H19 RNA, the expression of each allele was normalized to RPLPO and the values summed. This value correlated strongly (*r* = 0.86, *P* < 0.001) to our measurements of H19 performed by non‐allele specific PCR (Supplementary Figure 2) indicating that the approach was valid.

**Figure 5:**
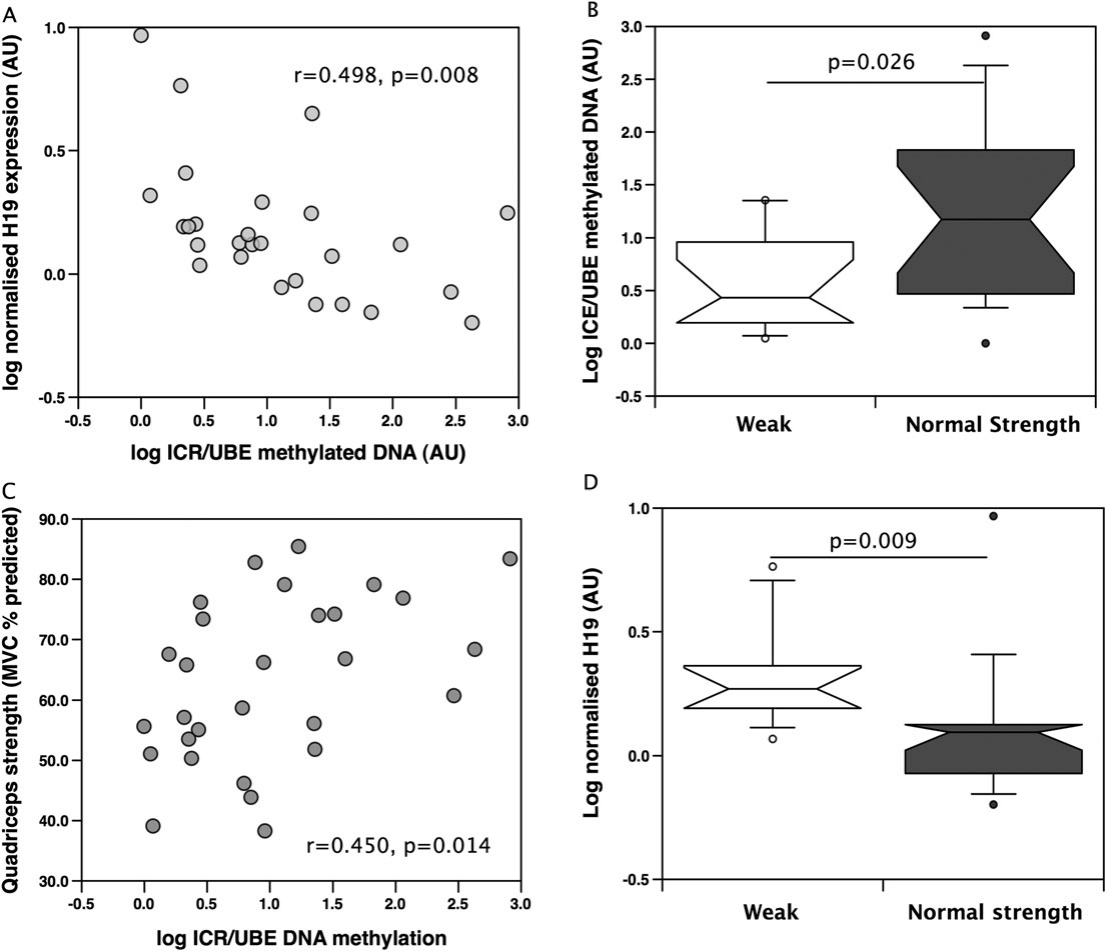
Methylation of the H19 locus is associated with H19 gene expression and strength. Methylated DNA was precipitated using the MeDiP kit as described in the Methods and input and precipitated DNA was quantified for the H19 ICR and for UBE2. ICR methylation is presented relative to the methylation of UBE2 (a sparsely methylated DNA region). H19 ICR methylation was inversely proportional to H19 gene expression (A), higher in weak patients compared to patients with normal strength (B) and correlated with quadriceps strength measured as % predicted (C). H19 expression was reduced in weak patients compared patients with normal strength (D).

To determine whether methylation regulates H19 expression in muscle, we used methylated DNA immunoprecipitation (MeDIP) followed by qPCR using muscle samples from 10 healthy controls, 14 normal FFMI patients, and 15 low FFMI patients. Methylation on the ICR of precipitated H19 DNA was quantified relative to a comparatively unmethylated gene UBE2B. There was no significant difference between relative ICR methylation between patients and controls. Consistent with methylation suppressing H19 expression, the ICR:UBE enrichment ratio was inversely correlated with H19 expression in the COPD patients (*r* = −0.498, *P* = 0.008, *Figure* 5A). Furthermore, the ICR/UBE methylation ratio was higher in patients with a normal strength compared to weak patients (*P* = 0.026, *Figure* 5B) and correlated with strength (*r* = 0.45, *P* = 0.014, *Figure* 5C) but not with FFMI. In this cohort of patients H19 correlated with strength (*r* = −0.45, *P* = 0.017) but not with FFMI and was different between patients with normal strength and weak patients (*P* = 0.009, *Figure* 5D). In the controls the association of relative ICR methylation with strength did not reach statistical significance (*r* = 0.57, *P* = 0.084) unless normalized to FFMI (*r* = 0.64, *P* = 0.045, Supplementary Figure 3). Similarly strength normalized for FFMI was also correlated with ICR methylation in the patients alone (*r* = 0.48, *P* = 0.009 ) and in the cohort as a whole (*r* = 0.45, *P* = 0.004). Methylation of the C19MC was not determined because these miRNAs are not expressed in differentiated cells.^36^, ^37^ As analysis of methylation in the biopsies would have profiled whole muscle, the majority of signal would have been from cells that did not express the miRNA rather than determining the effect of methylation on expression.

#### Are miR‐675 and miR‐519a markers of FFMI in the general population?

The association of miR‐675 and miR‐519a with FFFMI in COPD raises the possibility that they are markers of muscle mass in the general population rather than showing an adaptation to disease. We therefore determined the expression of both miRs in the quadriceps of a subset of individuals from the Hertfordshire Sarcopenia Study, which assessed muscle mass and function in healthy older adults. In this cohort neither miRNA was associated with FFMI or strength (measured as grip strength) or with physical capability marked by 3m walking time and 6m time to get up and go. Furthermore, there was no association of the expression of these miRNAs with birth weight. Comparison of the expression of miR‐675 and miR‐519a in people with a history of smoking and in non‐smokers also showed no difference.

### Altered regeneration in low FFMI patients

**Figure 6:**
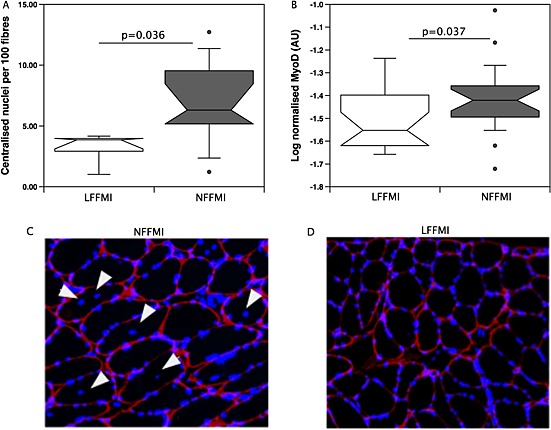
Reduced regeneration in the muscle of low fat‐free mass index patients. (A) sections of muscle were stained for laminin and DAPI as described in Methods. The number of centralized nuclei was determined by counting the number of transverse cut fibres with an intact laminin outline and the number of centralized nuclei in the whole section by an individual blinded to the group assignment of each image. Centralized nuclei are presented as number per 100 fibres. The number of centralized nuclei was higher in normal fat‐free mass index patients than in low fat‐free mass index patients. (C and D) Segments of representative images used in counting the number of centralized nuclei from a normal fat‐free mass index patient (C) and a low fat‐free mass index patient (D). White arrows indicate centralized nuclei. (B) MyoD mRNA expression was determined in the quadriceps of patients with chronic obstructive pulmonary disease as described in Methods. Normal fat‐free mass index patients expressed significantly more MyoD than low FFMI patients (*P* < 0.05).

The ability of miR‐675 to inhibit C2C12 cell proliferation (as previously described^38^ and as shown in supplementary information) raises the possibility that there is a difference in muscle regenerative activity in low FFMI patients and normal FFMI patients. We therefore determined the expression of MyoD, a marker of satellite cell activation, and the number centralized nuclei in samples from male patients with GOLD 3/4 COPD, the selection criteria for our original screen. MyoD was suppressed in the low FFMI patients compared to the normal FFMI patients (low FFMI = 0.031 ± 0.009 AU, normal FFMI = 0.041 ± 0.017 AU, *P* = 0.037, *Figure* 6A). The low FFMI patients also had fewer fibres with centralized nuclei indicating recent regeneration compared to normal FFMI patients (normal FFMI 6.9 ± 1.1 centralized nuclei/100 fibres vs. low FFMI 3.2 ± 0.6 centralized nuclei/100 fibres *P* = 0.036, *Figure* 6B–D). These findings are consistent with increased muscle regeneration occurring in normal FFMI patients compared to low FFMI patients. In patients in which centralized nuclei, miR‐675, and miR‐519a were all measured, miR‐675 was higher (low FFMI median was 2.4‐fold higher than the normal FFMI median, *P* = 0.014) and miR‐519a was lower (low FFMI median was 0.71 times the normal FFMI median, *P* = 0.008) in the low FFMI patients than in the normal FFMI patients.

## Discussion

The data presented show that a low FFMI and weakness in COPD patients are associated with increased expression of miR‐675 and H19 in the muscle and that muscle weakness correlates in these patients with relative methylation of the H19 ICR. Furthermore, the expression of miRNAs from the C19MC is suppressed in the muscle of male patients with a low FFMI and associated with FFMI. However, there was no association of miRNA expression with FFMI or strength in healthy individuals from the Herts Sarcopenia Study nor did smoking affect the expression of the miRNAs, suggesting that the associations of miRNA with FFMI and strength occur only on the background of the disease. Finally we confirm that there are fewer centralized nuclei in the muscle of low FFMI patients compared to patients with a normal FFMI raising the possibility that poor regeneration is contributory to the low FFMI phenotype. Together, these data indicate that the level of imprinted miRNAs contributes to the susceptibility to muscle wasting in the context of COPD, where the disease provides an atrophic stimulus that can be resisted in individuals to varying extents dependent on the expression of these miRNAs. The potential mechanisms by which these miRNAs contribute to the low FFMI phenotype are discussed separately below.

#### miR‐675‐H19

The H19‐IGF‐2 imprinted gene cluster is one of the best‐studied imprinted regions.^39^ In this cluster H19 is expressed from the maternal chromosome whereas IGF‐2 is expressed from the paternal chromosome with expression of H19 suppressed by methylation of an imprinting centre. H19 is a long intergenic non‐coding RNA the function of which appears to be to act as a source of miR‐675.^24^, ^38^ The fact that the observed associations of H19 with strength and FFMI were not as strong as those of miR‐675 is consistent with this suggestion. Previous studies of miR‐675 have shown that it inhibits cell proliferation and consistent with this observation we also found that miR‐675 could inhibit C2C12 proliferation. This inhibition of proliferation has been shown to occur at least in part through suppressing IGF1R,^38^ therefore increased miR‐675 in myofibres is likely to suppress hypertrophy.

Our data show that H19 and miR‐675 are expressed at low levels in proliferating cells but are markedly elevated in cells that have stopped proliferating and are differentiating (see Supporting Information). Recently it has also been shown that miR‐675 promotes myoblast differentiation^24^ by suppressing expression of SMAD‐1 and ‐5 leading to reduced BMP signalling as well as a suppression of cdc6 (a protein required for the G1‐S phase transition in the cell cycle). This study suggests three likely consequences of an elevated background of miR‐675 that would contribute to a low FFMI phenotype. First, in differentiated myofibres BMP signalling is pro‐hypertrophic,^40^and a reduction in BMP signalling has been shown to be pro‐atrophic.^40^ Second, as the analysis was performed in whole muscle homogenates we are unable to differentiate between changes in miR‐675 expression in myofibres and satellite cells. Consequently, if the increase in miR‐675 is not restricted to myofibres but is also present in myoblasts and satellite cells it will inhibit the expansion of the myoblast pool (by inhibiting cdc6 and reducing cell proliferation), and third, it will promote the early differentiation of these cells reducing the time available for myoblast proliferation and further restricting the pool of cells available for regeneration.

#### C19MC miRNAs

The C19 microRNA cluster is the largest cluster of miRNAs in the human genome but arose relatively late in evolution and is only found in primates.^21^ The miRNAs from this cluster are expressed in pluripotent cells and in the placenta but are not expressed in fully differentiated tissues.^36^, ^37^ It therefore seems likely that the transcripts that we detected arose from satellite cells rather than from the myofibres. Consistent with this suggestion the levels of C19MC miRNAs were low. Functionally the C19MC miRNAs have been shown to promote the pluripotent state in embryonic stem cells by targeting RBL‐2 and thereby relieving RBL‐2 dependent suppression of the expression of a methyltransferase DNMT3B a key regulator of embryonic differentiation.^41^ Knockdown of DNMT3B results in a reduction in embryonic stem cell survival and in the formation of embryonic stem cell‐derived clones *in vitro*. These data suggest that C19MC miRNAs may contribute to the normal FFMI phenotype by promoting stem cell survival.^36^, ^37^

It is important to note that the association of C19MC expression with FFMI was only evident in males with severe COPD. The lack of association of C19MCs in the cohort as a whole and in other groups individually may be because of confounding factors and/or statistical power as some of the sub‐groups were relatively small. It is also possible that it is only apparent in the severe subgroup as, in the absence of significant physiological stress because of severe disease, the requirement for regeneration will be limited to normal muscle cell turnover.

#### Imprinting and DNA methylation

Both C19MC and H19 expressions are regulated by imprinting.^42^ The C19MC locus is expressed from the paternal chromosome, whereas H19 is expressed from the maternal chromosome. In general, paternally imprinted genes promote growth whereas maternally imprinted genes inhibit growth, and our data are consistent with this observation. In muscle, imprinted genes are predominantly expressed in stem/progenitor cells in adult mice.^43^ This pattern of expression is consistent with an important functional role in the regulation of satellite cells and represents one way in which imprinted genes may contribute to the control of post‐natal skeletal muscle mass.^43^ As imprinting involves DNA methylation the involvement of imprinting suggests that DNA methylation makes an important contribution to the low FFMI and normal FFMI phenotypes. Methylation of the H19‐IGF‐2 ICR inhibits the expression of H19^44^ and our data showing an inverse correlation of H19 expression with relative H19‐IGF‐2 ICR methylation is consistent with this observation. Furthermore, our data showing that relative methylation of the H19‐IGF‐2 ICR is higher in normal FFMI patients and is correlated with muscle strength suggests that differences in DNA methylation of particular loci may contribute to the control of muscle function in adulthood in response to the stress of COPD.

Most studies of imprinted genes focus on development and the early post‐natal period. Consequently the association of H19 and miR‐675 with muscle mass and strength in adults has not previously been noted, and we did not find an association of miR‐675 with FFMI or strength in the Herts Sarcopenia Study participants. This observation suggests that the association is only present or detectable in patients with a disease that predisposes to muscle wasting. It is therefore possible that differences in the expression of this miRNA are only of functional significance when there is an increased atrophic drive and/or an increase in the demand for muscle synthesis.

#### Reduced regeneration in low FFMI patients

Consistent with the recent studies of Theriault *et al.* 45 who observed reduced centralized nuclei in patients with a mid thigh muscle cross‐sectional area < 70 cm^2^ compared to those with a mid thigh cross‐sectional area > 70 cm^2^ we found that patients with a low FFMI had reduced proportion of myofibres with centralized nuclei. This observation implies that a low level of regeneration contributes to the low FFMI phenotype. As described above both a low level of expression of C19MC miRNAs and an increased miR‐675 expression may contribute to this reduction in regeneration.

#### Critique of the method

The study is cross sectional, and as such the data can only demonstrate associations rather than causation. Nevertheless, our analysis of the muscle has highlighted a number of miRNAs with potential mechanistic relevance to the development of skeletal muscle dysfunction in COPD that warrant further investigation. Although one interpretation of our data is that there is a reduction in the number of satellite cells, we did not quantify satellite cell number directly because of the number and size of the samples available. Our analysis of centralized nuclei is consistent with the data of Theriault,^45^ and in their study they did not see a difference in satellite cell number between patients with a low or normal mid thigh cross‐sectional area suggesting that any differences are small. Consequently if there is a difference in satellite cell number it will require a large number of biopsies to identify it. It is also possible that the number of satellite cells does not change either because these cells have become senescent as suggested by Theriault *et al.* or because the effects of miR‐675 are on the proliferation of committed myoblasts rather than the number of satellite cells. Whether any such senescence results from high miR‐675 and/or low C19MC expression remains to be established.

### Clinical implications

There is significant variation in the amount or rate of loss of muscle mass in response to a range of diseases. In COPD, skeletal atrophy appears to be a significant factor in about 25% of patients that can start early in the disease.^3^ In a second example, we have recently shown that following major cardiac surgery approximately 50% of patients will lose more than 10% of their muscle mass in the following 7 days whereas the others will not.^46^ Furthermore, there is marked diversity in the response to treatment in patients with muscle wasting with only a subset responding well to pulmonary rehabilitation. This diversity hampers the development of methodologies to improve muscle mass. Understanding the basis for this variation in individual response to disease and treatment will help to identify those at greatest risk of wasting, to identify those likely to respond to therapies and to develop novel targeted therapies. Our data here suggest that expression levels of H19, miR‐675, and the C19MC miRNAs may contribute to this diversity of response and that DNA methylation is a potential contributor to this differential expression. It is possible therefore that measurement of these factors may help to identify sub‐groups of individuals likely to respond differently to anabolic therapies.

## Conclusions

Our data show that increased expression of miR‐675 and H19 are associated with a low FFMI in patients with COPD. Furthermore, increased expression of H19 is inversely correlated with relative methylation of the H19 ICR. In male patients with severe COPD, expression of the paternally expressed C19MC miRNAs was suppressed in patients with a low FFMI. We also confirmed that patients with a low FFMI had lower levels of muscle regeneration than patients with a normal FFMI. Together these data suggest that expression of imprinted miRNAs may contribute to the susceptibility to a low FFMI phenotype in COPD.

## Human studies

All subjects gave written informed consent; the COPD study protocol was approved by the Royal Brompton & Harefield NHS Trust Research Ethics Committee (Study numbers 06/Q0404/35 and 06/Q0410/54); the Herts Sarcopenia Study study protocol was approved by the Hertfordshire Research Ethics Committee (study 07/Q0204/68)

## Author contributions

PK and AL proposed the hypothesis for the study. AL, JL, AD, and SV performed the miRNA, mRNA DNA, and cell culture analysis supervised by PK. SAN collected the human COPD muscle biopsies supervised by MP. The Herts Sarcopenia Study study samples were collected by HPP under the supervision of AAS. PK wrote the first draft of the paper, and all authors contributed to the revision of the draft and the critical analysis of the project.

## Conflict of interest

Amy Lewis, Jen Lee, Anna Donaldson, Srividya Vaidyanathan, Holly Syddall, Avan Sayer, and Cyrus Cooper do not have any conflicts of interest. Paul Kemp has received research grant funding from GSK and Astra Zeneca, Samantha Natanek has received grant funding from GSK, and Michael Polkey has received research grant and/or consultancy funding from GSK, Lilly, Novartis, Biomarin Astra Zeneca, and Pfizer (paid to his institution).

## Supporting information



Supporting info item

Supporting info item

Supporting info item

Supporting info item

Supporting info item
