# Supplementary material for: Increased expression of H19/miR‐675 is associated with a low fat‐free mass index in patients with COPD
Source: J Cachexia Sarcopenia Muscle. 2016 Jan 5;7(3):330–44. doi: 10.1002/jcsm.12078 (PMC4863928; doi:10.1002/jcsm.12078)
Supplement: Supplementary file 1 — Supporting info item [file JCSM-7-330-s001.docx]

***Supplementary Methods***

*Cell culture:* C2C12 cells were maintained in culture as described[[33](#_ENREF_33)]. RNA was extracted using Trizol and mRNA and miRNAs were determined as described above. miR mimic transfection: cells were seeded into 96 well plates at 500 cells/well in octuplet. Each well was transfected the following day with 0.5 μl of 20 μM mimic with 0.5 μl lipofectamine 2000 in 50 μl Opti-MEM and 100 μl DMEM for 4h before the medium was replaced with DMEM supplemented with 10%FCS. Cells were quantified using CyQuant NF cell proliferation kit (Invitrogen) according to the manufacturer’s instructions.

#### Supplementary Results

**Mir-675 increases as myoblasts withdraw from the cell cycle and inhibits their proliferation *in vitro***

miR-675 has been associated with cell cycle inhibition [[34](#_ENREF_34)]. To determine whether it inhibited myoblast proliferation, we determined the expression profile of miR-675 and H19 in C2C12 cells during differentiation. The expression of cell cycle markers (cyclinE1, Chk1 and cdc25) was reduced, while those of differentiation (MHCs and myogenic bHLH proteins) were increased over the time course, consistent with previous studies of C2C12 differentiation [[35](#_ENREF_35), [36](#_ENREF_36)](Supplementary Fig. 4). Expression of H19, miR-675-3p, and -5p was also higher in myotubes compared to proliferating myoblasts (Supplementary Fig. 4).

To determine whether these miRNAs could directly inhibit myoblast proliferation, C2C12 cells were transfected with these miRNAs (675-5p and 3p) and proliferation was analysed. Transfection with miR-290 (a close murine equivalent to C19MC miRNAs that has the same AAGUGC seed sequence as miR-519a) or a scrambled miRNA served as negative controls and transfection with miR-1 as a positive control. miR-675 inhibited C2C12 cell proliferation to the same extent as miR-1. Neither the scrambled miRNA nor miR-290 suppressed cell proliferation (Supplementary Fig. 4) compared to untransfected cells.

**Supplementary table 1 Screen cohort**

|  | Control (n=7) | Normal FFMI (n=7) | Low FFMI (n=7) |
| --- | --- | --- | --- |
| Age (years) | 67± 11 | 64±4 | 68 ±6 |
| Smoking History^a^ (pack-year) | 6.25 (0, 24) | 49 (41, 60) * | 51 (32, 71) * |
| Weight ^a^ (kg) | 75.5 (74.1, 86.8) | 83.3 (72.1, 94.0) | \| 60.8 (54.5, 69.4)* \| \| --- \| |
| BMI^a^ (kg/m^2^) | 25.7 (24.8, 26.9) | 24.5 (24.4, 27.1) | 20.8 (19.9, 22.7) **, † |
| FFMI ^a^ (kg/m^2^) | 18.5 (17.5, 20.0) | 17.6 (16.4, 17.9) | 14.3 (13.9, 14.8)**, †† |
| FEV_1_^a^ (% pred) | 107.7 (104.0, 123.2) | 25.1 (22.0, 38.0)** | 31.5 (27.4, 41.5)** |
| RVTLC | 35± 4 | 61±9*** | 60±7*** |
| TLCO ^a^ (% pred) | 86.7 (84.5, 90.8) | 31.9 (28.2, 50.7)** | 29.0 (22.7, 43.3)** |
| 6 min walk (m) | 657± 102 | 367±159*** | 342±161*** |
| 6min walk % pred | 116±12 | 67±31*** | 63±25*** |
| pVO_2_ ^a^ (% pred) | 90 (84, 99) | 38 (33, 48)** | 44 (31, 46)** |
| SGRQ ^a^ | 1 (0, 8) | 53 (51, 62)** | 58 (51, 66)** |
| Quadriceps MVC (kg) | 39.9±6.6 | 32.5±7.9 | 27.0±9** |
| Quadriceps MVC (% pred) | 77 ± 15 | 62 ± 15 | 57±11* |
| Locomotion time^a^ (min/12 hr) | 90 (67, 115) | 30 (18, 59) | 61 (30, 77) |
| Movement time (as % of 12hrs) | 19.2±6.4 | 13.7±9.2 | 15.7±8.2 |
| Type I fibre % | 45.9±13.7 | 27.0±17.7* | 24.9±5.7** |
| Type IIA fibre % | 48.0±12.3 | 59.0±12.5 | 61.8±10.4 |
| Type IIX fibre % | 3.4±3.4 | 11.7±10.6** | 7.4±5.9** |

Definitions of abbreviations: ^a^= Not normally distributed, BMI = Body Mass Index, FFMI= Fat-Free Mass Index, FEV_1_ =Forced Expiratory Volume in 1 sec, RV =Residual volume, TLC = total lung capacity, TL_CO_= transfer coefficiant of the lung for CO, Pa_02_ = arterial oxygen partial pressure, Pa_C02_ = arterial carbon dioxide partial pressure, pred = predicted, MVC = Maximal Voluntary Contraction, SGRQ, St George’s respiratory questionnaire. Values are means ± SD for normally distributed variables and as median (interquartile range) for variables that were not normally distributed. Significance was calculated by t-Test for normally distributed variables and by Mann Whitney U test for variables that were not normally distributed. *(p < 0.05) ** (p = <0.01) ***(p= <0.001) low FFMI or normal FFMI vs control . †† (p=<0.01), ††† (P=<0.001) low FFMI vs normal FFMI

**Supplementary table 2: H19 cohort**

|  | Control (n=11) | Normal FFMI (n=34) | Low FFMI (n=22) |
| --- | --- | --- | --- |
| Sex (M, F) | 2, 9 | 19, 15 | 5, 17 |
| Age (years) | 68± 10 | 68±9 | 63 ±9 |
| Smoking History^a^ (pack-year) | 0 (0, 6) | 45 (34, 655) *** | 40 (30, 47) *** |
| Weight ^a^ (kg) | 64.7(61.0, 65.4.8) | 78.3 (73.9, 85.3)** | \| 59.7 (51.0, 65.1)††† \| \| --- \| |
| BMI^a^ (kg/m^2^) | 24.5 (23.6, 25.5) | 28.1 (25.9, 30.8)** | 21.7 (19.9, 23.5) *, ††† |
| FFMI ^a^ (kg/m^2^) | 15.3 (15.2, 15.9) | 17.7 (16.2, 18.5)** | 14.0 (13.4, 14.5)***, ††† |
| FEV_1_^a^ (% pred) | 110.5 (99.1, 113.0) | 58.5 (44.1, 71.9)*** | 43.8 (27.4, 59.0)***, †† |
| RVTLC | 39± 5 | 51±9*** | 57±8***,†† |
| TLCO ^a^ (% pred) | 82.5 (79.6, 95.1) | 52.5 (40.0, 61.8)** | 41.2 (26.1, 52.9)** |
| 6 min walk (m) | 603± 69 | 426±102*** | 374±136*** |
| 6min walk % pred | 127±12 | 90±19*** | 72±26***, †† |
| pVO_2_ ^a^ (% pred) | 97 (80, 110) | 60 (50, 70)** | 45 (34, 54)** |
| SGRQ ^a^ | 2 (1, 7) | 50 (37, 59)*** | 54 (47, 62)*** |
| Quadriceps MVC (kg) | 30.7±7.8 | 33.9±10.7 | 20.2±8.6*,††† |
| Quadriceps MVC (% pred) | 77 ± 17 | 72 ± 19 | 56±13**,††† |
| Locomotion time^a^ (min/12 hr) | 96 (59, 141) | 46 (36, 80)** | 40 (21, 55)*** |
| Movement time (as % of 12hrs) | 23.8±6.5 | 14.4±5.7** | 13.1±5.3*** |
| Type I fibre % | 53.8±12.5 | 32.6±10.6*** | 27.2±13.9*** |
| Type IIA fibre % | 42.0±11.6 | 56.4±11.0*** | 63.0±13.7*** |
| Type IIX fibre % | 1.9±3.1 | 5.1±5.7 | 5.3±5.3 |

Definitions of abbreviations: ^a^= Not normally distributed, BMI = Body Mass Index, FFMI= Fat-Free Mass Index, FEV_1_ =Forced Expiratory Volume in 1 sec, RV =Residual volume, TLC = total lung capacity, TL_CO_= transfer coefficiant of the lung for CO, Pa_02_ = arterial oxygen partial pressure, Pa_C02_ = arterial carbon dioxide partial pressure, pred = predicted, MVC = Maximal Voluntary Contraction, SGRQ, St George’s respiratory questionnaire. Values are means ± SD for normally distributed variables and as median (interquartile range) for variables that were not normally distributed. Significance was calculated by t-Test for normally distributed variables and by Mann Whitney U test for variables that were not normally distributed. *(p < 0.05) ** (p = <0.01) ***(p= <0.001) low FFMI or normal FFMI vs control . †† (p=<0.01), ††† (P=<0.001) low FFMI vs normal FFMI

**Supplementary table 3: Herts Sarcopenia Study cohort**

Definitions of abbreviations: ^a^= Not normally distributed, BMI = Body Mass Index, FFMI= Fat-Free Mass Index, FEV_1_ =Forced Expiratory Volume in 1 sec, TUG time = time to up and go.

|  | Whole cohort (n=67) | Non-smokers (n=32) | Current and ex smokers (n=35) |
| --- | --- | --- | --- |
| Weight ^a^ (kg) | 83.2 (72.8, 91.8) | 81.2 (70.9, 86.4) | \| 84.1 (80.0, 93.3) \| \| --- \| |
| BMI^a^ (kg/m^2^) | 27.1 (24.8, 29.1) | 25.8 (24.0, 28.7) | 27.4 (26.2, 29.3) |
| FFMI ^a^ (kg/m^2^) | 18.4 (17.4, 19.3) | 18.4 (17.1, 19.3) | 18.4 (17,8, 19.3) |
| FEV_1_^a^ (% pred) | 105.2 (98.2, 114.8) | 106.1 (103.1, 117.2) | 104.5 (92.3, 112.5) |
| TUG time (s) | 10.5 (9.2,12) | 10.1 (9.2, 10.9) | 10.9 (9.6, 12.25) |
| 3m walk time ^a^ (s) | 2.7 (2.5, 3.1) | 2.7 (2.5, 2.9) | 2.8 (2.5, 3.3) |
| Handgrip strength (kg) | 38.6±8.9 | 38.6±7.8 | 38.5±10.0 |
| Birth weight ^a^ (kg) | 3.2 (3.0, 3.9) | 3.2 (3.0, 4.0) | 3.2 (3.0, 3.9) |
| Log miR-675 (AU) | -2.8 ± 19 | -2.81 ± 0.58 | 2.83±0.54 |
| Log miR-519a (AU) | -4.22 ± 0.36 | -4.17 ± 0.28 | -4.26 ± 0.40 |

|  | Control (n=10) | Normal FFMI (n=14) | Low FFMI (n=15) |
| --- | --- | --- | --- |
| Age (years) | 66± 11 | 67±7. | 67 ±7 |
| Smoking History^a^ (pack-year) | 7 (0, 9) | 55 (40,72)*** | 37 (29, 50) ** |
| Weight ^a^ (kg) | 77.5 (74.0, 96.9) | 70.6 (63.2, 81.6) | \| 60.8 (54.7, 68.8)***,† \| \| --- \| |
| BMI^a^ (kg/m^2^) | 26.3 (24.7, 29.1) | 24.2 (22.8, 25.1) | 21.7 (19.6, 22.1)**, †† |
| FFMI ^a^ (kg/m^2^) | 19.0 (17.5, 20.5) | 16.9 (16.4, 17.1)* | 14.5 (14.5, 15.1)***, ††† |
| FEV_1_^a^ (% pred) | 107.7 (101.4, 111.1) | 28.5 (24.1, 35.9)*** | 31.6 (27.0, 39.5)*** |
| RVTLC | 34± 4 | 63±7*** | 60±9*** |
| TLCO ^a^ (% pred) | 90.5 (86.7, 97.3) | 40.3 (28.3, 46.2)*** | 39.3 (27.1, 54.6)*** |
| 6 min walk (m) | 630± 101 | 332±119*** | 360±151*** |
| 6min walk % pred | 120±14 | 72±23*** | 80±27*** |
| pVO_2_ ^a^ (% pred) | 96 (89, 104) | 42 (34, 46)*** | 44 (31, 49)*** |
| SGRQ ^a^ | 3 (0, 8) | 50 (42, 61)*** | 58 (49, 66)*** |
| Quadriceps MVC (kg) | 42.5±9.3 | 32.6±8.3** | 28.1±7.3*** |
| Quadriceps MVC (% pred) | 82 ± 20 | 67 ± 15* | 59±12** |
| Locomotion time^a^ (min/12 hr) | 85 (61, 97) | 37 (20, 41)** | 47 (20, 71)* |
| Movement time (as % of 12hrs) | 17.0±4.9 | 11.0±5.3 | 12.7±6.4 |
| Type I fibre % | 53.1±18.1 | 29.2±14.3*** | 25.9±13.6 *** |
| Type IIA fibre % | 39.8±17.4 | 62.7±8.6*** | 64.1±15.7*** |
| Type IIX fibre % | 3.6±3.6 | 5.6±8.9 | 5.7±5.1 |

**Supplementary Table 4 Physiological parameters for the Methylation cohort**

Definitions of abbreviations: ^a^= Not normally distributed, BMI = Body Mass Index, FFMI= Fat-Free Mass Index, FEV_1_ =Forced Expiratory Volume in 1 sec, RV =Residual volume, TLC = total lung capacity, TL_CO_= transfer coefficiant of the lung for CO, Pa_02_ = arterial oxygen partial pressure, Pa_C02_ = arterial carbon dioxide partial pressure, pred = predicted, MVC = Maximal Voluntary Contraction, SGRQ, St George’s respiratory questionnaire. Values are means ± SD for normally distributed variables and as median (interquartile range) for variables that were not normally distributed. Significance was calculated by t-Test for normally distributed variables and by Mann Whitney U test for variables that were not normally distributed. *(p < 0.05) ** (p = <0.01) ***(p= <0.001) low FFMI or normal FFMI vs control . †† (p=<0.01), ††† (P=<0.001) low FFMI vs normal FFMI

**Supplementary table 5 Physiological Parameter centralized nuclei cohort**

|  | Normal FFMI (n=10) | Low FFMI (n=5) |
| --- | --- | --- |
| Sex (M, F) | 10,0 | 5,0 |
| Age (years) | 63±5 | 68 ±6 |
| Smoking History^a^ (pack-year) | 55 (38, 68) | 75 (68, 80) |
| Weight ^a^ (kg) | 79.8 (72.6, 90.8) | \| 64.2 (60.8.5, 71.7), \| \| --- \| |
| BMI^a^ (kg/m^2^) | 25.2 (24.3, 26.7) | 22.0 (20.3, 23.4) |
| FFMI ^a^ (kg/m^2^) | 16.8 (16.4, 17.9) | 14.5 (14.5, 14.6) †† |
| FEV_1_^a^ (% pred) | 33.7 (25.7, 49.6) | 27.6 (27.6, 45.2) |
| RVTLC | 59±9 | 62±4 |
| TLCO ^a^ (% pred) | 44.3 (29.2, 52.4) | 26.2 (19.1, 29.9) |
| 6 min walk (m) | 387±162 | 357±118 |
| 6min walk % pred | 70±29 | 65±18 |
| pVO_2_ ^a^ (% pred) | 41 (32, 49) | 34 (31, 45) |
| SGRQ ^a^ | 55 (50, 67) | 45(41, 60) |
| Quadriceps MVC (kg) | 34.2.7±9.8 | 27.4±7.2 |
| Quadriceps MVC (% pred) | 66±15 | 56±7 |
| Locomotion time^a^ (min/12 hr) | 37 (23, 66) | 61 (52, 82) |
| Movement time (as % of 12hrs) | 14±8 | 16±6 |
| Type I fibre % | 28.6 ± 18.1 | 23.4 ± 8.4 |
| Type IIA fibre % | 59.0 ± 15.1 | 60.6 ± 11.6 |
| Type IIX fibre % | 9.5 ± 9.4 | 10.6 ± 8.6 |

Definitions of abbreviations: ^a^= Not normally distributed, BMI = Body Mass Index, FFMI= Fat-Free Mass Index, FEV_1_ =Forced Expiratory Volume in 1 sec, RV =Residual volume, TLC = total lung capacity, TL_CO_= transfer coefficiant of the lung for CO, Pa_02_ = arterial oxygen partial pressure, Pa_C02_ = arterial carbon dioxide partial pressure, pred = predicted, MVC = Maximal Voluntary Contraction, SGRQ, St George’s respiratory questionnaire. Values are means ± SD for normally distributed variables and as median (interquartile range) for variables that were not normally distributed. Significance was calculated by t-Test for normally distributed variables and by Mann Whitney U test for variables that were not normally distributed. *(p < 0.05) ** (p = <0.01) ***(p= <0.001) low FFMI or normal FFMI vs control . †† (p=<0.01), ††† (P=<0.001) low FFMI vs normal FFMI

**Supplementary Table 6. Low FFMI associated miRNAs in the quadriceps muscle of COPD patients**

|  | Median fold change | | | P value | | |
| --- | --- | --- | --- | --- | --- | --- |
| miRNA | LFFMI vs NFFMI | LFFMI vs cont | NFFMI vs cont | LFFMI vs NFFMI | LFFMI vs cont | NFFMI vs cont |
| miR-519c-3p | 0.137 | 0.220 | 1.611 | 0.000 | 0.008 | 0.393 |
| miR-518a-3p | 0.265 | 0.354 | 1.338 | 0.000 | 0.038 | 0.171 |
| miR-517a | 0.366 | 0.438 | 1.197 | 0.000 | 0.000 | 0.079 |
| miR-517c | 0.386 | 0.568 | 1.471 | 0.000 | 0.004 | 0.068 |
| miR-519a | 0.451 | 0.438 | 0.971 | 0.000 | 0.002 | 0.300 |
| miR-512-3p | 0.523 | 0.519 | 0.991 | 0.000 | 0.005 | 0.325 |
| miR-525-3p | 0.341 | 0.438 | 1.286 | 0.001 | 0.003 | 0.524 |
| miR-518b | 0.428 | 0.398 | 0.931 | 0.001 | 0.013 | 0.385 |
| miR-519d | 0.511 | 0.515 | 1.009 | 0.001 | 0.004 | 0.422 |
| miR-517b | 0.137 | 0.103 | 0.748 | 0.002 | 0.004 | 0.398 |
| miR-523 | 0.314 | 0.553 | 1.760 | 0.002 | 0.036 | 0.139 |
| miR-101 | 0.531 | 0.527 | 0.993 | 0.002 | 0.001 | 0.862 |
| miR-515-5p | 0.334 | 0.370 | 1.106 | 0.003 | 0.017 | 0.342 |
| miR-518e | 0.541 | 0.518 | 0.959 | 0.003 | 0.011 | 0.562 |
| miR-520g | 0.132 | 0.106 | 0.801 | 0.004 | 0.003 | 0.751 |
| miR-522 | 0.280 | 0.256 | 0.912 | 0.004 | 0.006 | 0.544 |
| miR-32 | 0.505 | 0.359 | 0.711 | 0.004 | 0.000 | 0.118 |
| miR-15a | 0.343 | 0.577 | 1.680 | 0.005 | 0.001 | 0.071 |
| miR-26b | 0.579 | 0.525 | 0.906 | 0.005 | 0.001 | 0.253 |
| miR-342-3p | 0.632 | 0.658 | 1.041 | 0.007 | 0.000 | 0.788 |
| miR-342-5p | 0.263 | 0.144 | 0.547 | 0.008 | 0.010 | 0.651 |
| miR-126 | 0.734 | 0.591 | 0.805 | 0.008 | 0.001 | 0.255 |
| miR-340 | 0.771 | 0.570 | 0.739 | 0.010 | 0.001 | 0.082 |
| miR-489 | 0.521 | 0.656 | 1.259 | 0.011 | 0.018 | 0.268 |
| miR-140-5p | 0.687 | 0.602 | 0.876 | 0.011 | 0.001 | 0.492 |
| miR-186 | 0.745 | 0.672 | 0.903 | 0.013 | 0.010 | 0.599 |
| miR-518d-5p | 0.235 | 0.251 | 1.070 | 0.015 | 0.006 | 0.675 |
| miR-148b | 0.635 | 0.568 | 0.896 | 0.015 | 0.014 | 0.623 |
| miR-139-5p | 0.724 | 0.617 | 0.851 | 0.017 | 0.004 | 0.216 |
| miR-190 | 0.489 | 0.325 | 0.664 | 0.018 | 0.001 | 0.151 |
| miR-146a | 0.615 | 0.754 | 1.226 | 0.018 | 0.026 | 0.784 |
| miR-195 | 0.770 | 0.645 | 0.837 | 0.018 | 0.004 | 0.102 |
| miR-374a | 0.516 | 0.440 | 0.851 | 0.019 | 0.004 | 0.166 |
| miR-148a | 0.665 | 0.531 | 0.799 | 0.024 | 0.011 | 0.437 |
| let-7f | 0.798 | 0.653 | 0.818 | 0.024 | 0.015 | 0.703 |
| miR-193a-3p | 0.462 | 0.336 | 0.727 | 0.025 | 0.006 | 0.388 |
| miR-1 | 0.679 | 0.775 | 1.141 | 0.029 | 0.041 | 0.662 |
| miR-208b | 0.642 | 0.375 | 0.584 | 0.030 | 0.002 | 0.567 |
| miR-628-5p | 0.589 | 0.862 | 1.464 | 0.031 | 0.029 | 0.327 |
| miR-454 | 0.481 | 0.444 | 0.923 | 0.034 | 0.008 | 0.475 |
| miR-125a-5p | 0.703 | 0.676 | 0.961 | 0.037 | 0.036 | 0.633 |
| miR-30b | 0.848 | 0.780 | 0.920 | 0.037 | 0.008 | 0.621 |
| miR-98 | 0.610 | 0.649 | 1.063 | 0.038 | 0.017 | 0.513 |
| miR-590-5p | 0.794 | 0.639 | 0.805 | 0.039 | 0.003 | 0.080 |
| miR-361-5p | 0.706 | 0.811 | 1.148 | 0.041 | 0.029 | 0.329 |
| miR-424# | 3.893 | 28.569 | 7.340 | 0.009 | 0.000 | 0.220 |
| miR-675 | 8.506 | 7.002 | 0.823 | 0.008 | 0.030 | 0.182 |

miRNAs that were suppressed in LFFMI patients compared to NFFMI patients are shown above the line in order of statistical significance, those increased in LFFMI patients compared to LFFMI patients are shown below the line in order of statistical significance.

**Supplementary Figure legends**

**Supplementary Figure 1: Expression of miR-675 is correlated with H19 in the muscle of COPD patients and controls.**

The expression of miR-675 and H19 were determined by qPCR as described and compared in the individuals where both determinations were possible. The expression of miR-675 was positively correlated with H19 expression in these samples. Patients are shown as grey circles and controls are shown as black circles

**Supplementary Figure 2: Comparison of two methods of determining H19 expression.**

H19 expression was determined using Sybr Green and by combining the results of two independent measures using the SNP assay. There was a tight correlation of the values obtained by these two methods. Patients are shown as grey circles and controls are shown as black circles

**Supplementary Figure 3. Relative DNA methylation is associated with MVC normalized for FFMI in COPD patients and controls**

Methylated DNA was precipitated using the MeDiP kit as described in the Methods and input and precipitated DNA was quantified for the H19 ICR and for UBE2. (A) Relative ICR methylation was not different between patients and controls. Relative ICR methylation was associated with QMVC normalized for FFMI in patient (B) and in controls (C).

Supplementary Figure 4. miR- 675 and H19 are increased in differentiating myoblasts *in vitro*

RNA was extracted from C2C12 and differentiating C2C12 cells 120h prior to (proliferating cells) and 48h, 96h, and 144h after the induction of differentiation by placing the cells into medium supplemented with 2% horse serum as described in Methods. QPCR was used to determine the expression of myosin heavy chains (MHCI, MHCIIA, MHCIIX and MHCIIB, A), myogenic transcription factors (myoD, myogenin and myf5, B) and cell cycle genes (Chk1, CDC25A and cyclin E1, C). The expression of H19, miR-675 is shown in D. Data are shown as mean +/- SEM from quadruplicates. (E) C2C12 cells were transfected with miR mimics for miR-675-3p, miR-675-5p, miR-290, a scrambled control or no oligonucleotide (lipo) as described in Methods. The cells were counted after 24, 48 or 96 h in growth medium. MiR-675-3p and miR-675-5p inhibited cell proliferation compared to scrambled or no transfected controls whereas miR-290 did not affect cell number. The data were from 2 separate experiments each performed in 8 biological repeats at each time point.
