# Supplementary figures and images for: Increased expression of H19/miR‐675 is associated with a low fat‐free mass index in patients with COPD
Source: J Cachexia Sarcopenia Muscle. 2016 Jan 5;7(3):330–44. doi: 10.1002/jcsm.12078 (PMC4863928; doi:10.1002/jcsm.12078)

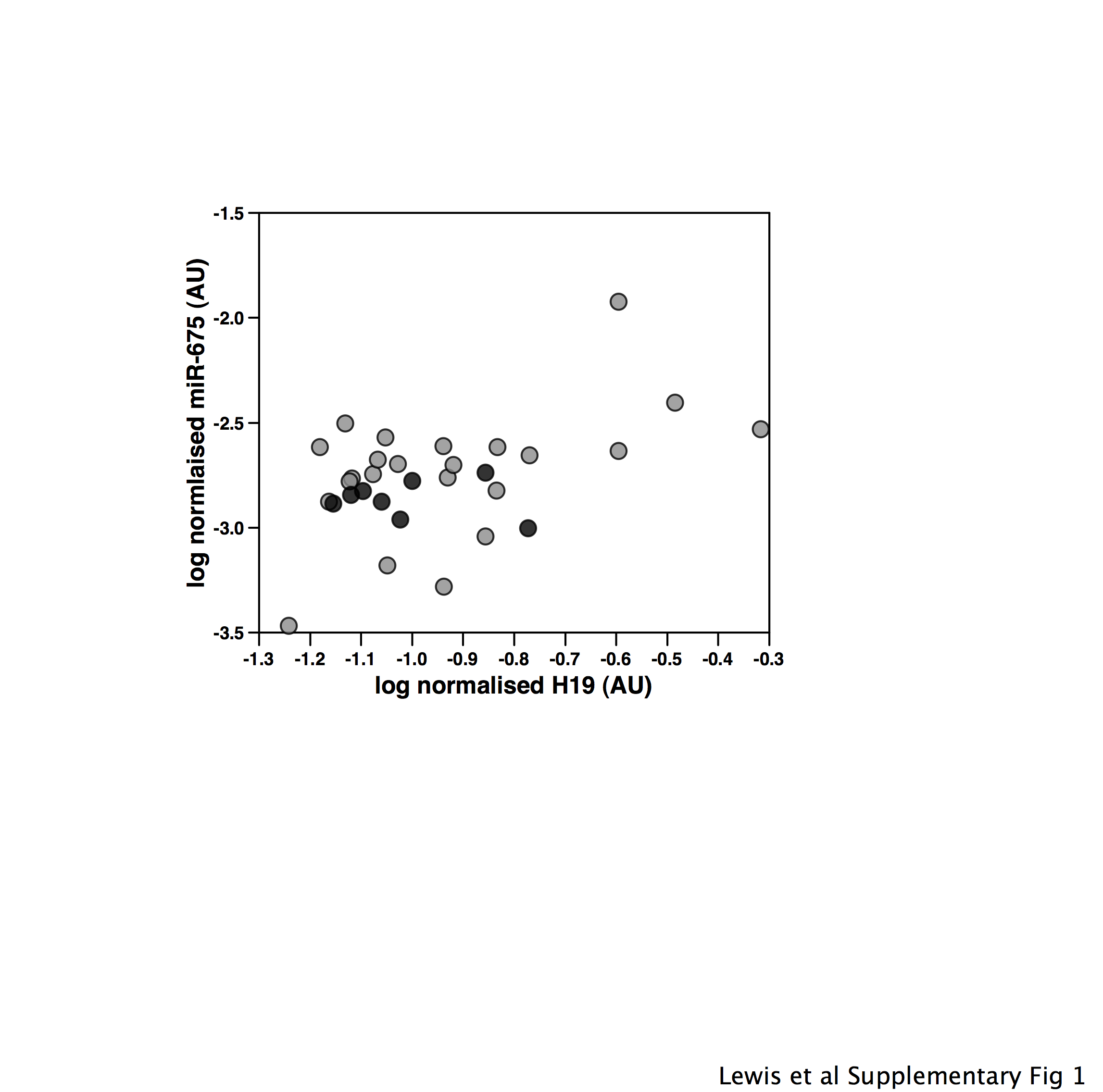

Supplement: Supplementary file 2 — Supporting info item [file JCSM-7-330-s002.tiff]

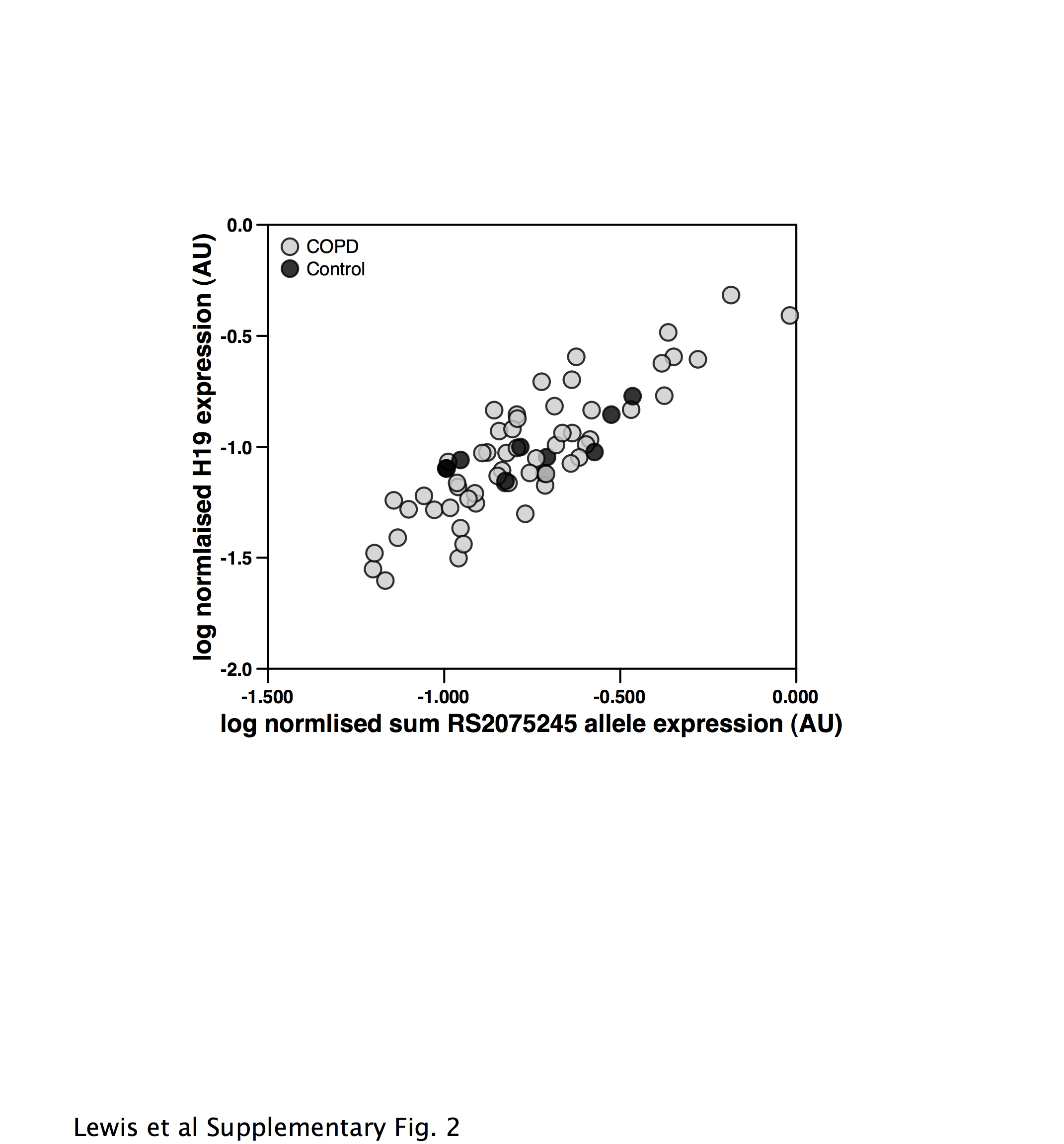

Supplement: Supplementary file 3 — Supporting info item [file JCSM-7-330-s003.tiff]

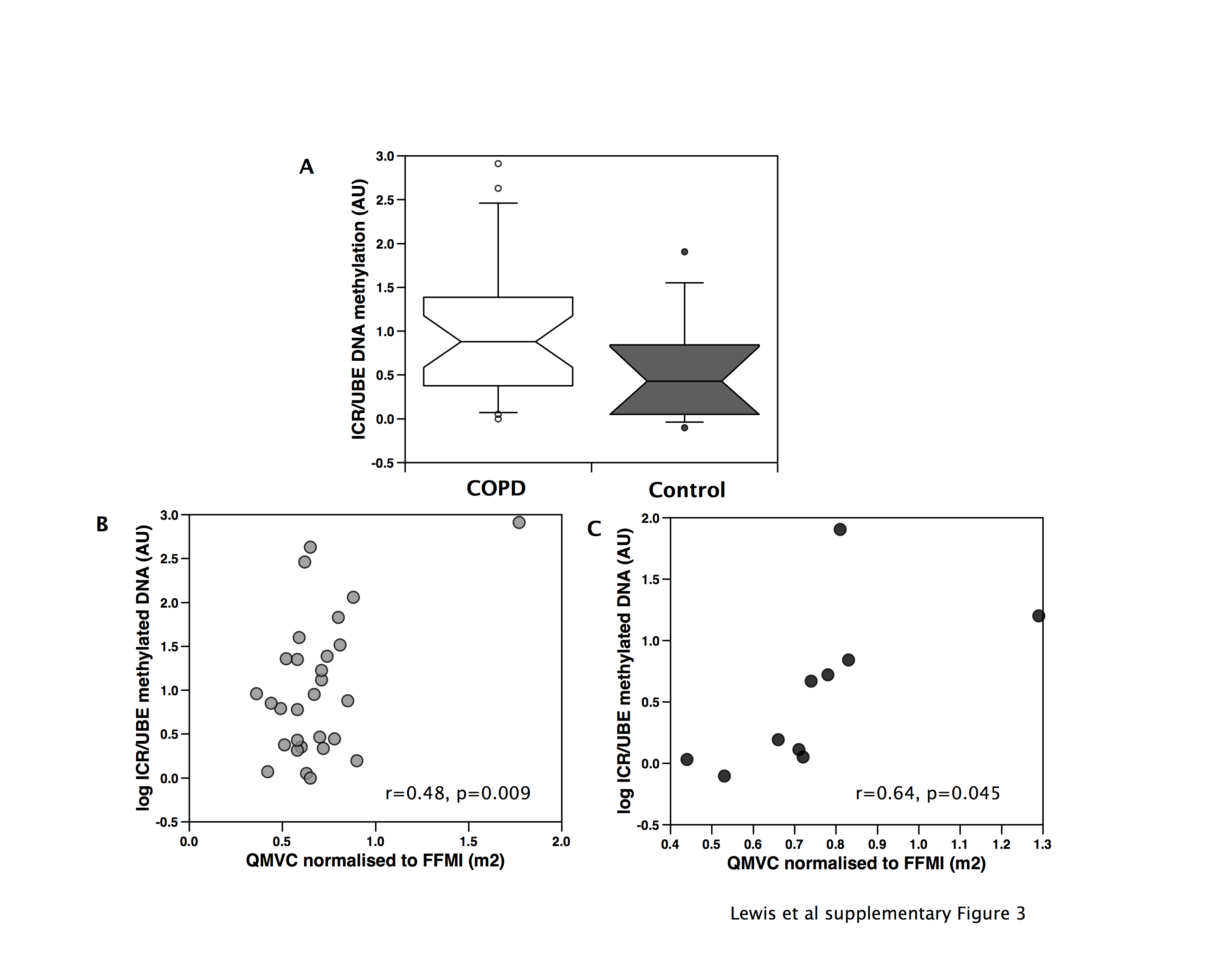

Supplement: Supplementary file 4 — Supporting info item [file JCSM-7-330-s004.tiff]

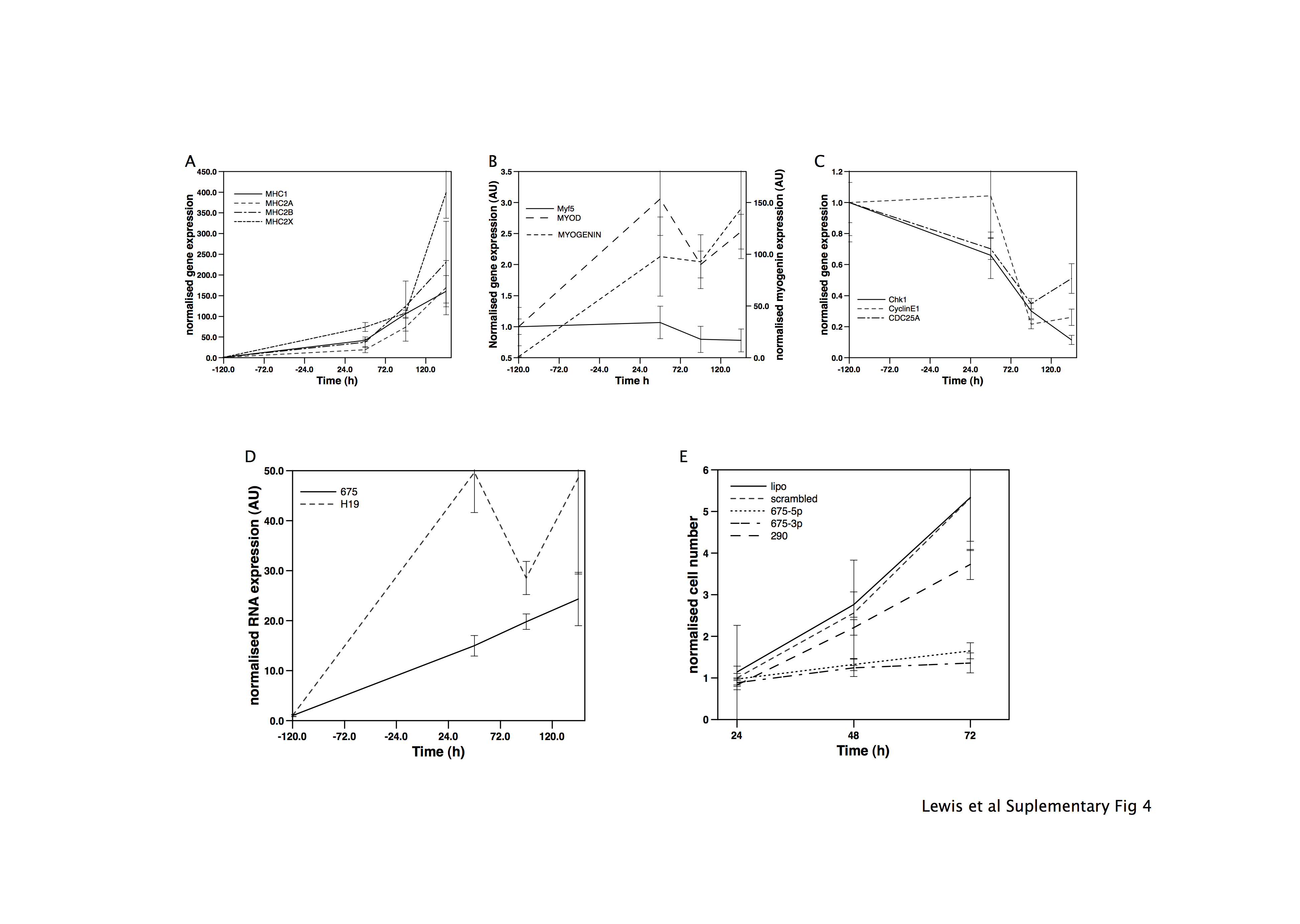

Supplement: Supplementary file 5 — Supporting info item [file JCSM-7-330-s005.tiff]
